# Quantitative MRI assessment of left ventricular filling: associations with left atrial and pulmonary artery dimensions in a population-based study

**DOI:** 10.3389/fcvm.2026.1902569

**Published:** 2026-08-05

**Authors:** Charlotte Wintergerst, Roberto Lorbeer, Susanne Rospleszcz, Esther Askani, Blerim Mujaj, Bernard E. Bulwer, Annette Peters, Holger Schulz, Christopher L. Schlett, Fabian Bamberg, Ricarda von Krüchten

**Affiliations:** 1 Department of Diagnostic and Interventional Radiology, Medical Center - University of Freiburg, Faculty of Medicine, University of Freiburg, Freiburg, Germany; 2 Department of Radiology, University Hospital, LMU Munich, Munich, Germany; 3 German Centre for Cardiovascular Research (DZHK) Partner Site Munich Heart Alliance, Munich, Germany; 4 General Practice, Aalst, Belgium; 5 Cardiovascular Imaging Core Laboratory, Cardiovascular Division, Department of Medicine, Brigham and Women’s Hospital and Harvard Medical School, Boston, MA, United States; 6 Institute of Epidemiology, German Research Center for Environmental Health, Helmholtz Zentrum Munich, Neuherberg, Germany; 7 Department of Epidemiology, Institute for Medical Information Processing, Biometry, and Epidemiology, Ludwig-Maximilians-University Munich, Munich, Germany; 8 German Center for Diabetes Research (DZD), Partner Site Neuherberg, Neuherberg, Germany; 9 German Center for Cardiovascular Disease Research (DZHK e.V.), Munich, Germany; 10 Comprehensive Pneumology Center Munich (CPC-M), German Center for Lung Research, Munich, Germany

**Keywords:** left atrium, LV diastolic dysfunction, magnetic resonance imaging, population imaging, subclinical

## Abstract

**Introduction:**

Left ventricular diastolic dysfunction is associated with left atrial remodeling and alterations of the pulmonary circulation. However, whether these interconnected changes across the cardiopulmonar*y* axis can be simultaneously characterized using a non-dedicated whole-body MRI examination in individuals free of overt cardiovascular disease remains unclear.

**Methods:**

339 subjects (mean age 56.4 years; 43.4% female) without overt cardiovascular disease were included. Linear regression models, adjusted for age, sex, and cardiovascular risk factors, explored the association of Left ventricle (LV) filling rates with left atrium (LA) size and function, and mean pulmonary artery (MPA).

**Results:**

Average maximal LA was 19.6 cm^2^, minimal LA 11.8 cm^2^, area-based LA function 39.8%, non-electrocardiogram-synchronized LA size 16.8 cm^2^, LV early filling rate 227.1 mL/s, late filling rate 238.4 mL/s, and MPA diameter 2.67 cm. Higher early and late LV filling rates were associated with larger maximal LA size across all models (*β* = 2.30; *β* = 1.00), minimal LA size (*β* = 1.41; *β* = 0.77), and non-electrocardiogram-synchronized LA size (*β* = 1.86; *β* = 0.90, all *p* < 0.001, fully adjusted model). Higher early LV filling rates, but not higher late LV filling rates, were associated with larger MPA diameter across all models (*β* = 0.06, *p* = 0.03, fully adjusted model).

**Conclusion:**

In individuals free of overt cardiovascular disease, MRI-derived LV filling dynamics were associated with structural alterations of the left atrium and pulmonary vasculature, suggesting that changes across the cardiopulmonar*y* axis may already be detectable by whole-body MRI before clinical disease becomes apparent.

## 1 Introduction

Whole-body MRI examinations are increasingly performed for various clinical and research-driven indications (1, 2). These examinations enable a rapid, non-invasive, comprehensive thoraco-abdominal evaluation of the patient, potentially facilitating the detection of subtle cardio-pulmonary alterations whilst being free of ionizing radiation. Heart failure (HF) affects an estimated 1%–2% of adults, although this may be underestimated (3, 4). The risk factors and clinical presentation of heart failure with preserved ejection fraction (HFpEF) and heart failure with reduced ejection fraction overlap substantially. However, the underlying pathophysiological mechanisms and response to treatment differ significantly (5). The pathophysiology of HFpEF, previously known as diastolic heart failure, which affects approximately 50% of patients with HF (6), is characterized by abnormal diastolic function due to left ventricular (LV) wall stiffness and impaired relaxation. This results in altered LV filling dynamics that are reflected in the left atrium (LA), the pulmonary vasculature, and the right heart. Therefore, elevated LA pressures and LA remodeling, as well as right ventricular (RV) dysfunction, are key features of HFpEF (7, 8). The presence of overt symptoms defines severe LV diastolic dysfunction and HFpEF, but subclinical LV diastolic dysfunction is far more prevalent and is an independent predictor of all-cause mortality, even in asymptomatic individuals (9, 10). Although altered diastolic filling and elevated left atrial and pulmonary capillary wedge pressure are keys to diagnosing HFpEF, precise measurement requires invasive catheter-based measurements (11). Echocardiography is widely used for the non-invasive assessment of diastolic function. However, this modality is operator-dependent and provides a limited assessment of the pulmonary vasculature (12). Cardiac MRI provides a reproducible, operator-independent, and radiation-free assessment of cardiac function, enabling quantitative evaluation of ventricular filling dynamics and non-invasive assessment of the left atrium and pulmonary vasculature. Little is known about how early alterations in LV filling dynamics are simultaneously associated with atrial remodeling and pulmonary vascular changes in individuals free of overt cardiovascular disease. Furthermore, whether these interconnected components of the cardiopulmonar*y* axis can be assessed by a single whole-body MRI examination without a dedicated protocol remains largely unexplored. The purpose of our study was thus to evaluate the relationship between MR-based left ventricular filling rate, structural and functional parameters of the LA, and pulmonary artery dimensions in a population without cardiovascular disease using whole-body MRI examination.

## 2 Materials and methods

### 2.1 Study population and ethics approval

**Figure 1:**
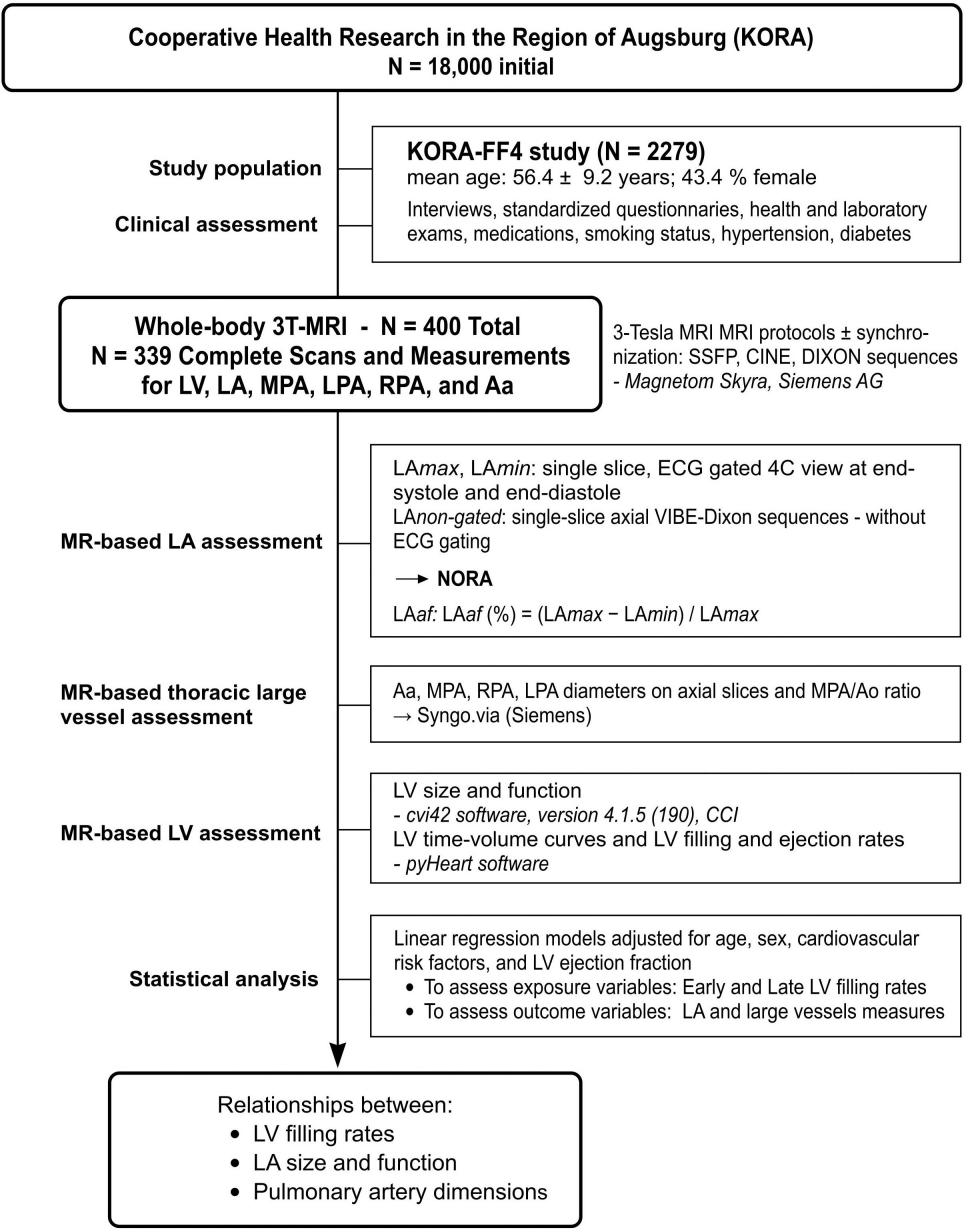
Flow chart of the study protocol. Among 18,000 participants in the Cooperative Health Research in the Region of Augsburg (KORA), 400 underwent whole-body MRI. Of these, 339 had complete scans and measurements and were included in the present analysis. Aa, ascending aorta; CCI, Circle Cardiovascular Imaging; ECG, electrocardiogram; KORA, Cooperative Health Research in the Region of Augsburg; LA, left atrium; LAaf, left atrium area fraction; LAmax, maximum left atrium area; LAmin, minimum left atrium area; LA non-gated, left atrium area derived from axial non-gated sequences; LPA, left pulmonary artery; LV, left ventricle; MPA, main pulmonary artery; MPA/Ao ratio, main pulmonary artery/ascending aorta ratio; MRI, magnetic resonance imaging; RPA, right pulmonary artery; SSFP, steady-state free precession.

Our research was carried out as part of the prospective cohort within the Cooperative Health Research in the Region of Augsburg (KORA) (13, 14). KORA is a longitudinal, population-based epidemiological study, initially enrolling 18,000 participants across four distinct subgroups (S1-S4), followed by periodic health assessments (13). The KORA-FF4 study (*n* = 2279) is one of these follow-ups, including participants from the S4 group. In total, 400 individuals from this KORA-FF4 cohort agreed to and underwent whole-body MRI between June 2013 and September 2014 as part of a KORA-MRI study (14, 15). Participants were excluded from the study based on several criteria, such as prior cardiovascular conditions (e.g., heart attack, stroke, or vascular surgery), age over 72, non-MRI-compatible implants, pregnancy, breastfeeding, claustrophobia, kidney failure, or known allergies to gadolinium (14). Details regarding the study protocol are depicted in Figure 1. The KORA-MRI study received approval from the Institutional Research Ethics Board of the Medical Faculty at Ludwig-Maximilian University in Munich and was conducted in accordance with the ethical guidelines outlined in the Declaration of Helsinki on Human Research (16).

### 2.2 Clinical assessment

The KORA FF4 assessments included interviews, standardized questionnaires, health examinations, laboratory studies, and medication documentation and were conducted at the KORA study facility (13, 14). Body surface area (BSA) was determined using the Du Bois formula (BSA = 0.007184 * height^0.725 * weight^0.425). Smoking status was classified into three categories: never-smoker, former smoker, and current smoker. Hypertension was defined either as systolic blood pressure ≥ 140 mmHg or diastolic blood pressure ≥ 90 mmHg, as well as the use of antihypertensive medications. Diabetes diagnosis followed the criteria established by the WHO in 1998 (17); Prediabetes was defined as a normal fasting glucose level with a 2-hour oral glucose tolerance test serum glucose between 140 and 200 mg/dL, or a fasting glucose of 110–125 mg/dL with a normal 2-hour value. Newly diagnosed diabetes was identified by a 2-hour oral glucose tolerance test glucose >200 mg/dL and/or a fasting glucose >125 mg/dL. Subjects with fasting glucose <110 mg/dL and 2-hour oral glucose tolerance test glucose <140 mg/dL were classified as healthy controls (14). Alcohol consumption was reported in interviews.

### 2.3 Whole-body magnetic resonance imaging

The study involved a comprehensive whole-body MRI scan conducted with a 3-Tesla MRI (Magnetom Skyra, Siemens AG, Healthcare Sector, Erlangen, Germany). The MRI protocol for whole-body imaging has been thoroughly described before (14). To evaluate the maximum and minimum LA dimensions and the LV, unenhanced electrocardiography (ECG)-synchronized Cine- Steady-state free precession sequences were used with a breath-hold technique. The parameters for these sequences were: slice thickness of 8 mm, voxel dimensions of 1.5 × 1.5 mm^2^, field of view (FOV) of 297 × 360 mm, matrix size of 240 × 160, repetition time (TR) of 29.97 ms, echo time (TE) of 1.46 ms, and a flip angle of 62°. For LA area measurements without cardiac gating, axial sequences were used with the following parameters: slice thickness of 1.7 mm, voxel size of 1.7 × 1.7 mm^2^, FOV of 488 × 716 mm, matrix of 256 × 256, TR of 4.06 ms, TE ranging from 1.26 to 2.49 ms, and a flip angle of 9° (14). Imaging parameters for the analysis of the ascending aorta (Aa) diameter and pulmonary artery (PA) were the following: slice thickness 3 mm, voxel size 1.5 × 1.5 mm2, FOV 297 × 360 mm, matrix 320 × 195, TR/TE 4.1/1.23; 2.46; 3.69; 4.92; 6.15; 7.38, flip angle 9° (18).

### 2.4 MR-based LA assessment

The analysis of the LA size was conducted using the “NORA” medical platform (https://www.nora-imaging.com). Manual segmentation involved identifying the maximum and minimum LA areas (LAmax, LAmin) on a single slice of gated 4-chamber view CINE images, measured at end-systole and end-diastole, respectively. In addition, LA area quantification without ECG gating, was performed manually on a single axial slice at the level of the LV outflow tract and mitral valve using opposed-phase volumetric interpolated breath-hold examination (VIBE)-Dixon sequences. Measurements excluded the pulmonary veins but included the LA appendage. The non-electrocardiogram-synchronized LA area measurement has previously been described and evaluated in the same study population by Kulka et al. (19). Instead of using volume-based LA ejection fraction, we introduced an area-based metric called the left atrial area fraction calculated using the formula: left atrial area fraction (%) = (LAmax−LAmin)/LAmax (19).

### 2.5 MR-based thoracic large vessel assessment

The analysis of the Aa, main pulmonary artery (MPA) diameter, and right pulmonary artery and left pulmonary artery (LPA) was carried out using the medical software Syngo.via (Siemens, Healthcare, Erlangen, Germany). The outer diameter of the Aa was measured at the level of the right pulmonary artery on axial slices in an anterior-posterior orientation. For the assessment of the MPA diameter the height of the pulmonary artery branch was determined from axial images (18). A reference line, approximately 3 cm (±0.1 cm) in length, was drawn along the vessel's trajectory, following validated CT protocols (20, 21). The diameter of the vessel was then measured perpendicular to this reference line. right pulmonary artery and LPA were measured on the axial slice exhibiting their maximum diameter. The main pulmonary artery/ascending aorta ratio was defined by measuring the diameter of the Aa at the same slice as the MPA; furthermore, a cut-off value for the main pulmonary artery/ascending aorta ratio was set at 1.0 (22).

### 2.6 MR-based left ventricle assessment

The left ventricle size and function assessment was performed using Circle Cardiovascular Imaging (CCI) Inc.'s cvi42 software version 4.1.5 (190), (Calgary, Alberta, Canada). Initially, the left ventricular size and function were assessed automatically. Subsequently, manual corrections were made as needed (23). The in-house software pyHeart, designed to display LV time-volume curves, was utilized to quantify LV filling and ejection rates (24, 25). Reader certification demonstrated good reproducibility of LV volumes and ejection fraction (relative difference <5%), as previously reported (26). Intra-reader certification of MRI-derived LV filling rates in 39 participants showed a mean relative difference of <1%.

### 2.7 Statistical analysis

MRI-based cardiac and pulmonary data, pulmonary function measurements, and clinical characteristics of participants are presented as arithmetic means with standard deviations (SD) for continuous variables or as counts and percentages for categorical variables. Differences between women and men were tested by t-test or chi^2^-test. Linear regression models were used to assess the associations between left ventricular early and late filling rates (exposure variables), left atrial measurements, and thoracic large vessel diameters (outcome variables). The models provided *β*-coefficients with 95% confidence intervals (CI). Initial models were adjusted for sex and age, with subsequent adjustments for BSA, smoking status, hypertension, and diabetes. Further adjustments included cholesterol levels, triglycerides, hypertension medication, and lipid-lowering medication, with the final adjustment accounting for LV ejection fraction. Multivariate regression analyses were further performed separately for female and male participants. Statistical significance was defined as a two-sided *p*-value < 0.05. All analyses were conducted using Stata 18.0 (Stata Corporation, College Station, TX, USA).

## 3 Results

Out of the 400 participants who underwent whole-body MRI scans, 339 had complete and adequate measurements for the LA, LV, pulmonary artery, and the Aa. Table 1 shows the clinical characteristics and the MRI results of the study population. The study population's mean age was 56.4 ± 9.2 years, with 147 females (43.4%) and 192 males (56.6%). The average BSA was measured at 1.94 m^2^, and the mean body mass index (BMI) was recorded at 28.0 kg/m^2^. On average, 120 individuals (35.4%) had never smoked; 147 individuals (43.4%) were Ex-smokers, and 72 Individuals (21.2%) were current smokers. Hypertension was identified in 115 individuals (33.9%), and a total of 83 participants (24.5%) reported using antihypertensive medication. The majority of participants (207, 61.1%) were classified as having no diabetes, while prediabetes was identified in 90 participants (26.6%), and 42 individuals (12.4%) had a diabetes diagnosis. On average, the participants consumed 17,80 grams of alcohol per day. The lipid profile results showed a mean total cholesterol level of 218.7 mg/dL, high-density lipoprotein cholesterol level of 61.8 mg/dL, low-density lipoprotein cholesterol level of 140.8 mg/dL, and a mean triglyceride level of 132.4 mg/dL. Additionally, 35 participants (10.3%) were on lipid-lowering medications.

**Table 1:** Characteristics of the sample a) clinical characteristics and b) results of the MRI-examination.

**a)**				
Characteristics of the sample	*N* = 339	Female	Male	*p*
		*N* = 147	*N* = 192	
Age (years)	56.4 (±9.2)	56.4 (±9.2)	56.4 (±9.2)	0.963
Body surface area (m^2^)	1.94 (±0.22)	1.78 (±0.17)	2.07 (±0.17)	<0.001
BMI (kg/m^2^)	28.0 (±4.8)	27.5 (±5.5)	28.4 (±4.2)	0.091
Smoking status				0.139
Never Smoker	120 (35.4%)	59 (40.1%)	61 (31.8%)	
Ex-Smoker	147 (43.4%)	55 (37.4%)	92 (47.9%)	
Current smoker	72 (21.2%)	33 (22.5%)	39 (20.3%)	
Pack years	13.4 (±18.6)	8.6 (±12.4)	17.1 (±21.6)	<0.001
Hypertension	115 (33.9%)	41 (27.9%)	74 (38.5%)	0.040
Systolic BP (mmHg)	121.1 (±17.1)	113.5 (±14.4)	126.8 (±16.7)	<0.001
Diastolic BP (mmHg)	75.4 (±10.2)	72.1 (±8.5)	78.0 (±10.7)	<0.001
Antihypertensive medications	83 (24.5%)	39 (26.5%)	44 (22.9%)	0.443
Diabetes Status				0.016
No Diabetes	207 (61.1%)	102 (69.4%)	105 (54.7%)	
Prediabetes	90 (26.6%)	33 (22.5%)	57 (29.7%)	
Diabetes	42 (12.4%)	12 (8.2%)	30 (15.6%)	
HbA1c (%)	5.59 (±0.75)	5.59 (±0.59)	5.59 (±0.75)	0.946
Alcohol use, g/day	17.8 (±21.8)	8.4 (±11.9)	25.0 (±24.7)	<0.001
Total cholesterol (mg/dL)	218.7 (±37.3)	221.2 (±35.5)	216.8 (±38.6)	0.282
HDL (mg/dL)	61.8 (±17.8)	70.7 (±17.6)	55.1 (±14.8)	<0.001
LDL (mg/dL)	140.8 (±33.5)	138.6 (±32.8)	142.5 (±34.1)	0.280
Triglycerides (mg/dL)	132.4 (±86.1)	102.7 (±42.7)	155.2 (±102.6)	<0.001
Lipid- lowering medications	35 (10.3%)	15 (10.2%)	20 (10.4%)	0.949

**b)**				
MR measurements	*N* = 339	Female	Male	*p*
		*N* = 147	*N* = 192	
Vessel diameter				
Main pulmonary artery (cm)	2.67 (±0.34)	2.67 (±0.35)	2.67 (±0.33)	0.891
Right pulmonary artery (cm)	1.84 (±0.26)	1.81 (±0.26)	1.87 (±0.26)	0.018
Left pulmonary artery (cm)	1.94 (±0.24)	1.92 (±0.24)	1.97 (±0.24)	0.055
Main pulmonary artery/ Ascending aorta	0.91 (±0.15)	0.97 (±0.15)	0.87 (±0.13)	<0.001
MPA/Aa > 1	80 (23.6%)	55 (37.4%)	25 (13%)	<0.001
Ascending aorta (cm)	2.97 (±0.4)	2.79 (±0.36)	3.10 (±0.37)	<0.001
LA MR measurements				
LAmax (cm^2^)	19.6 (±4.5)	19.3 (±4.0)	19.7 (±4.9)	0.368
Lamin (cm^2^)	11.8 (±3.5)	11.2 (±3.1)	12.3 (±3.7)	0.007
LA area fraction (%)	39.8 (±9.2)	42.3 (±8.3)	37.9 (±9.4)	<0.001
LA non-gated (cm^2^)	16.8 (±4.0)	15.3 (±3.4)	17.9 (±4.0)	<0.001
LV MR measurements				
LV ejection fraction (%)	69.2 (±7.8)	71.1 (±6.9)	67.7 (±8.2)	<0.001
LV early diastolic filling rate (ml/s)	227.1 (±115.9)	230.8 (±104.8)	224.2 (±123.9)	0.603
LV late diastolic filling rate (ml/s)	238.4 (±144)	234.9 (±137.1)	241.2 (±149.3)	0.690

Data are means with standard deviation or number with percentage; *p*-values are from t-test or chi^2^-test.

Aa, ascending aorta; BMI, body mass index; CI, confidence interval; HbA1c, glycated hemoglobin A1c; HDL, high-density lipoprotein; LA, left atrium; LAaf, left atrium area fraction; LAmax, maximum left atrium area; LAmin, minimum left atrium area; LA non-gated, left atrium area derived from axial, non-gated sequences; LDL, low-density lipoprotein; LV, left ventricle; MPA, main pulmonary artery; MR, magnetic resonance.

The average LAmax was 19.6 cm^2^, the LAmin measured 11.8 cm^2^, the left atrial area fraction was 39.8%, and LA non-gated measured 16.8 cm^2^. LV measurements revealed a mean ejection fraction of 69.2%, an early diastolic filling rate of 227.1 mL/s, and a late diastolic filling rate of 238.4 mL/s. The mean diameters of the pulmonary arteries were 2.67 cm for the MPA, 1.84 cm for the right pulmonary artery, and 1.94 cm for the LPA.

### 3.1 Association of diastolic filling rates with left atrial size and function

**Figure 2:**
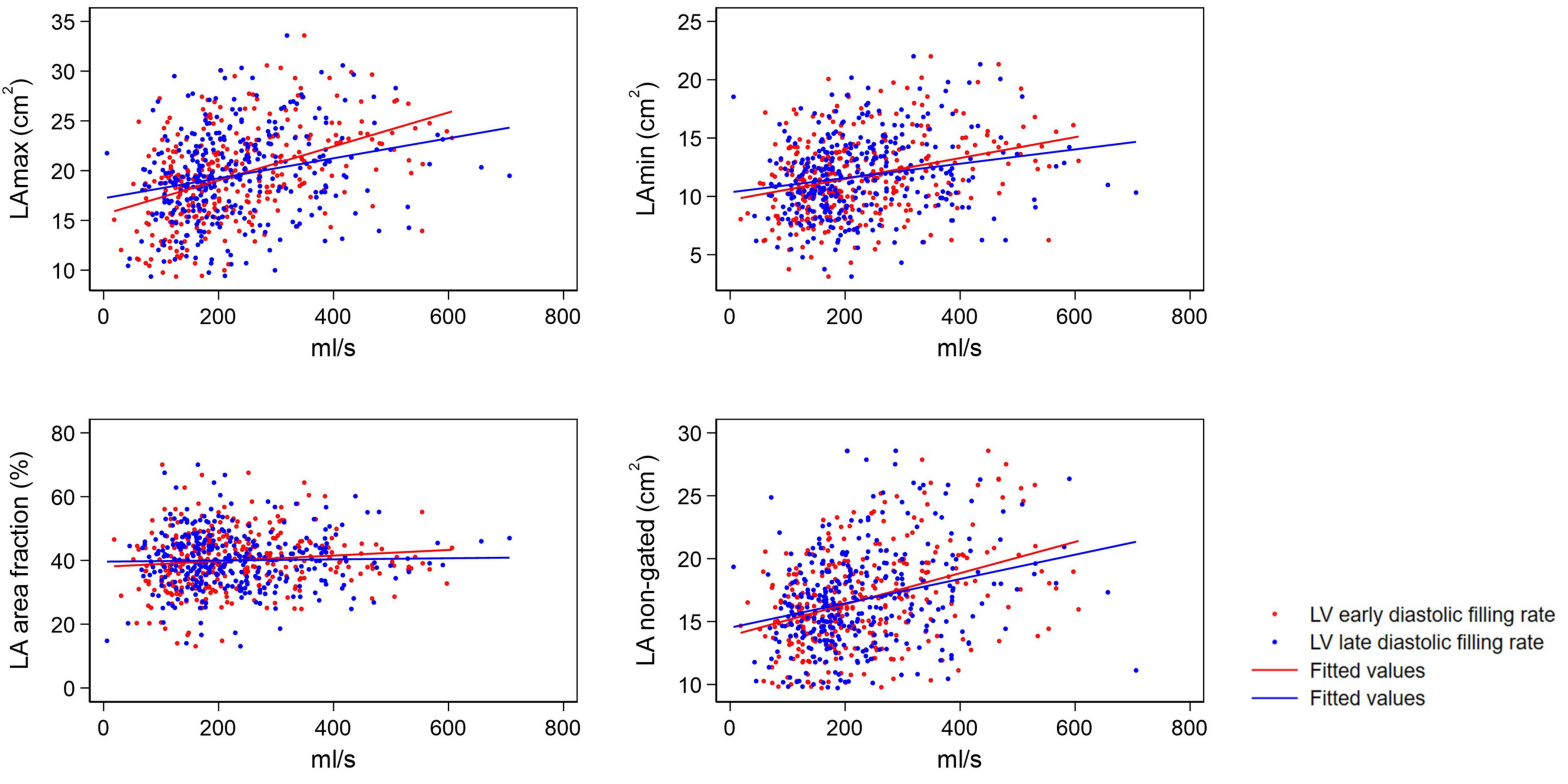
Scatter plot delineating the relationship between left atrium measurements and early and late left ventricular filling rate. Individual dots represent observed data points. The locally weighted regression line represents fitted values estimated from the observed data, illustrating the association between exposure and outcome. LA, left atrium; LAmax, maximum left atrium area; LAmin, minimum left atrium area; LA area fraction, left atrium area fraction calculated as (LAmax-LAmin)/LAmax; LA non-gated, left atrium area derived from axial, non-gated sequences.

Figure 2 depicts the relationship between early and late diastolic filling rates and LA size and function. We found a positive association between early diastolic filling rate and LA max (*β* = 2.16), LA min (*β* = 1.28), and LA non-gated (*β* = 1.77; all *p* ≤ 0.001) in model 1 adjusted for sex and age (model 1 in Table 2a). The association remained significant after further adjustment for other risk factors in models 2, 3, 4, and 5, with LA max (*β* = 2.30), LA min (*β* = 1.41), and LA non-gated (*β* = 1.86; all *p* ≤ 0.001, model 5) (Table 2a). Late diastolic filling rate was significantly positively associated with LA max (*β* = 1.13; *β* = 1.00), LA min (*β* = 0.86; *β* = 0.77), and LA non-gated (*β* = 1.06; *β* = 0.90, all *p* ≤ 0.001, model 1; model 5) in model 1 adjusted for age and sex and remained significant in models 2,3,4, and the fully adjusted model 5 (Table 2a). Neither early nor late diastolic filling rate was significantly associated with LA area fraction across all models (Table 2a).

**Table 2:** Association between LV early and late diastolic filling rate, LA parameters (a) and pulmonary artery dimensions (b).

**a)**										
Parameter per SD	Model 1	*P* value	Model 2	*P* value	Model 3	*P* value	Model 4	*P* value	Model 5	*P* value
LAmax (cm^2^)										
LV early diastolic filling rate (ml/s)	2.16 (1.70; 2.62)	**<0** **.** **001**	2.15 (1.70; 2.60)	**<0**.**001**	2.30 (1.83; 2.76)	**<0**.**001**	2.3 (1.82; 2.77)	**<0**.**001**	2.30 (1.82; 2.77)	**<0**.**001**
LV late diastolic filling rate (ml/s)	1.13 (0.65; 1.61)	**<0**.**001**	1.07 (0.59; 1.54)	**<0**.**001**	1.01 (0.53; 1.49)	**<0**.**001**	1.00 (0.50; 1.49)	**<0**.**001**	1.00 (0.50; 1.49)	**<0**.**001**
LAmin (cm^2^)										
LV early diastolic filling rate (ml/s)	1.28 (0.91; 1.64)	**<0**.**001**	1.26 (0.91; 1.62)	**<0**.**001**	1.41 (1.05; 1.78)	**<0**.**001**	1.41 (1.03; 1.78)	**<0**.**001**	1.41 (1.03; 1.78)	**<0**.**001**
LV late diastolic filling rate (ml/s)	0.86 (0.50; 1.22)	**<0**.**001**	0.80 (0.45; 1.16)	**<0**.**001**	0.78 (0.42; 1.14)	**<0**.**001**	0.76 (0.40; 1.13)	**<0**.**001**	0.77 (0.40; 1.14)	**<0**.**001**
LA area fraction (%)										
LV early diastolic filling rate (ml/s)	0.48 (−0.50; 1.47)	0.333	0.51 (−0.46; 1.48)	0.305	0.22 (−0.79; 1.24)	0.666	0.28 (−0.75; 1.31)	0.594	0.28 (−0.75; 1.32)	0.588
LV late diastolic filling rate (ml/s)	−0.45 (−1.40; 0.49)	0.345	−0.35 (−1.29; 0.59)	0.466	−0.43 (−1.38; 0.52)	0.374	−0.38 (−1.35; 0.59)	0.443	−0.41 (−1.38; 0.55)	0.401
LA non-gated (cm^2^)										
LV early diastolic filling rate (ml/s)	1.77 (1.39; 2.15)	**<0**.**001**	1.75 (1.39; 2.11)	**<0**.**001**	1.88 (1.51; 2.25)	**<0**.**001**	1.86 (1.48; 2.24)	**<0**.**001**	1.86 (1.49; 2.23)	**<0**.**001**
LV late diastolic filling rate (ml/s)	1.06 (0.66; 1.45)	**<0**.**001**	0.96 (0.59; 1.34)	**<0**.**001**	0.93 (0.55; 1.31)	**<0**.**001**	0.88 (0.49; 1.27)	**<0**.**001**	0.90 (0.51; 1.29)	**<0**.**001**

**b)**										
Parameter per SD	Model 1	*P* value	Model 2	*P* value	Model 3	*P* value	Model 4	*P* value	Model 5	*P* value
LV early diastolic filling rate (ml/s)	0.07 (0.03; 0.10)	**0**.**001**	0.07 (0.03; 0.10)	**0**.**001**	0.07 (0.03; 0.11)	**0**.**001**	0.06 (0.02; 0.10)	**0**.**003**	0.06 (0.02; 0.10)	**0**.**003**
LV late diastolic filling rate (ml/s)	0.05 (0.01; 0.08)	**0**.**012**	0.04 (0.01; 0.08)	**0**.**025**	0.04 (0; 0.07)	0.057	0.03 (−0.01; 0.07)	0.125	0.03 (−0.01; 0.07)	0.112
Right pulmonary artery (cm)										
LV early diastolic filling rate (ml/s)	0.03 (0; 0.06)	0.067	0.03 (0; 0.05)	0.070	0.03 (0; 0.06)	**0**.**040**	0.03 (0; 0.05)	0.100	0.03 (0; 0.06)	0.101
LV late diastolic filling rate (ml/s)	0.03 (0; 0.06)	**0**.**043**	0.02 (0; 0.05)	0.077	0.03 (0; 0.05)	0.073	0.02 (−0.01; 0.05)	0.174	0.02 (−0.01; 0.05)	0.175
Left pulmonary artery (cm)										
LV early diastolic filling rate (ml/s)	0.07 (0.04; 0.09)	**<0**.**001**	0.07 (0.04; 0.09)	**<0**.**001**	0.07 (0.04; 0.09)	**<0**.**001**	0.06 (0.04; 0.09)	**<0**.**001**	0.06 (0.04; 0.09)	**<0**.**001**
LV late diastolic filling rate (ml/s)	0.05 (0.03; 0.08)	**<0**.**001**	0.05 (0.03; 0.07)	**<0**.**001**	0.05 (0.03; 0.07)	**<0**.**001**	0.05 (0.02; 0.07)	**<0**.**001**	0.05 (0.02; 0.07)	**<0**.**001**

Data are β-coefficients with 95% confidence interval from linear regression models or odds ratios (OR) with 95% confidence interval from logistic regression models.

Model 1: Adjusted for sex and age.

Model 2: Model 1 + BSA.

Model 3: Model 2 + smoking status, hypertension status, diabetes status.

Model 4: Model 3 + total cholesterol, triglycerides, hypertension medication, lipid-lowering medication.

Model 5: like Model 4 + adjusted for LVEF.

BSA, body surface area; LA, left atrium; LAaf, left atrium area fraction; LAmax, maximum left atrium area; LAmin, minimum left atrium area; LA non-gated, left atrium area derived from axial, non-gated sequences; LDL, low-density lipoprotein; LV, left ventricle; LVEF, left ventricular ejection fraction; MPA, main pulmonary artery.

Bold values indicate statistical significance (*p* < 0.05).

### 3.2 Association of diastolic filling rates with pulmonary artery dimensions

**Figure 3:**
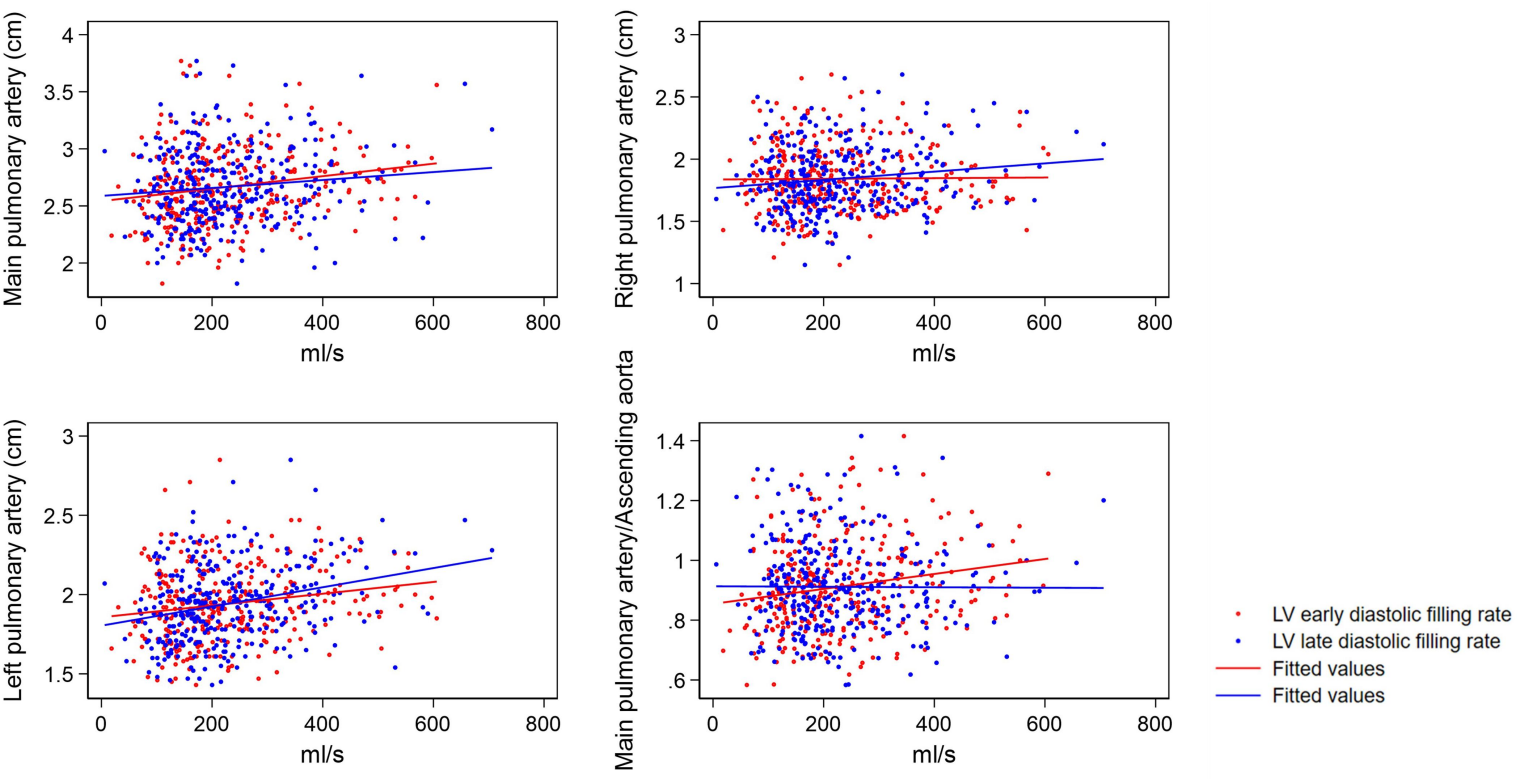
Scatter plot delineating the relationship between pulmonary vessel size and early and late diastolic filling rates. Individual dots represent observed data points. The locally weighted regression line represents fitted values estimated from the observed data, illustrating the association between exposure and outcome. LV, left ventricle.

Figure 3 shows the relationship between pulmonary vessel size and early and late diastolic filling rates. Across the models, higher early diastolic filling rates were significantly associated with larger MPA size (*β* = 0.06, *p* = 0.003), LPA (*β* = 0.06, *p* ≤ 0.001), and main pulmonary artery/ascending aorta ratio (*β* = 0.02, *p* = 0.038, model 5) (Table 2b, Supplementary Table S1). Late diastolic filling rate was positively associated with MPA size in model 1 adjusted for sex and age, and model 2, further adjusted for BSA (*β* = 0.05, *p* = 0.012, and *β* = 0.4, *p* = 0.25), and with right pulmonary artery in model 1 (*β* = 0.03, *p* = 0.043). Across all models, late diastolic filling rate was positively associated with LPA (*β* = 0.05, *p* ≤ 0.001, model 5) (Table 2b). No association was observed between late diastolic filling rate and the ratio of main pulmonary artery/ascending aorta (Supplementary Table S1).

### 3.3 Association of diastolic filling rates with left atrial size and function according to sex

Sex-stratified analyses are presented in Supplementary Tables S2a, S3a. In both women and men, higher early diastolic filling rates were consistently associated with larger LAmax, LAmin, and non-gated LA area across all models. Higher late diastolic filling rates were consistently associated with these LA measurements in men, whereas in women these associations were limited to the minimally adjusted models.

### 3.4 Association of diastolic filling rates with pulmonary artery dimensions according to sex

Sex-stratified analyses are presented in Supplementary Tables S2b, S3b. In women, higher early diastolic filling rates were associated with larger MPA and LPA diameters, whereas no significant associations were observed for late filling rates. In men, early filling rates were associated with LPA diameter, while late filling rates were associated with MPA and LPA diameters. Associations with the MPA/Aa ratio are shown in Supplementary Table S1.

## 4 Discussion

In this population-based MRI study, we found that higher early and late LV diastolic filling rates were significantly associated with larger LA size, and early LV diastolic filling rate with larger MPA size in participants free of manifest cardiovascular disease. To our knowledge, our study is the first to simultaneously assess LV filling rates, LA size, and function, as well as PA dimensions in a population-based sample using whole-body MRI. Despite relying on surrogate parameters, MRI was able to detect subtle morphological and functional alterations in individuals free of overt disease manifestations, underscoring the potential of non-invasive whole-body MRI to identify early, subclinical changes of the cardiopulmonar*y* axis, even without using a designated protocol. While alterations in these individual components have previously been described in LVDD and HFpEF, their interrelationship within the cardiopulmonar*y* axis has not been well characterized in individuals free of overt cardiovascular disease. Understanding these early associations may provide insights into the development of cardiopulmonary remodeling before the onset of clinical disease. Importantly, our findings describe associations across the physiological and subclinical spectrum, providing insights into early cardiopulmonary remodeling.

The pathophysiological changes in symptomatic HFpEF and LV diastolic dysfunction (LVDD) remain an active area of research. In the stepwise approach for the diagnosis of symptomatic HFpEF as recommended by the European Society of Cardiology, both increased LA size and abnormalities in diastolic filling are major diagnostic features of HFpEF (8).

Previous echocardiographic studies have shown an independent correlation between LA size, LVDD, and LVDD severity (27–29). These findings are consistent with our study, in which early and late LV filling rates were significantly associated with both the minimum and maximum LA size derived from ECG-gated sequences, and LA measurements from non-ECG-gated axial MR sequences, across all models. We did not detect any association between LV filling rates and area-based LA function, contrary to published ultrasound studies that suggested impaired LA function to be a superior marker of LVDD than LA size (27). This may be due to early-stage diastolic alterations in our population or intermodal differences between echocardiographic and MR-based assessment of LA function. Future studies combining cardiac MRI and echocardiography are warranted to further evaluate the agreement between CMR-derived markers and established echocardiographic measures of diastolic function.

The cardiopulmonar*y* axis plays a significant role in the development of HFpEF, with elevated PCWP being a diagnostic marker of HFpEF (11, 30). MPA diameter is an established but indirect imaging marker associated with pulmonary vascular remodeling. We found a significant association across all models of early LV filling rate to MPA-diameter, and an association of late LV filling rate to MPA-diameter in the models adjusted for sex, age, and BSA. Furthermore, a significant association between early and late LV filling rate and LPA diameter could be observed. While both cardiac and pulmonary disease share several established risk factors with an overlap of cardiac and pulmonary symptoms, significant interdependence exists between heart and lung functions. Progressive LV wall stiffness and impaired LV relaxation are associated with altered LV filling dynamics accompanied by LA remodeling and an increase in LA size, changes in the pulmonary circulation, and elevated pulmonary capillary resistance (30, 31). Our results may help to elucidate the subclinical associations of variations in LV filling dynamics within the LV-LA-pulmonary vessel axis that can be simultaneously assessed by whole-body MRI, even in individuals with subclinical disease.

In contrast to heart failure with reduced ejection fraction, HFpEF is more prevalent in females than in males; however, men seem to be more prone to die from HFpEF (32). Women are more likely to develop a hypertrophied, stiff, and non-dilated LV, a characteristic feature of HFpEF (33). A previous study using invasive hemodynamic assessments has revealed that men tend to have more severe pulmonary vascular disease, characterized by higher diastolic pressure gradients and a lower right ventricular ejection fraction (32). In our study, subtle sex-related differences were observed, primarily in the associations with pulmonary vessel size; however, these exploratory findings require confirmation in larger studies specifically designed to assess sex-specific effects.

The volume of the LA can be accurately measured using cardiac MRI; however, these measurements require a short-axis stack through the LA or gated CINE images in two planes (34, 35). As both routine cardiac MRI and the MRI acquisition protocol of the present study primarily focused on ventricular volumes and function, the second imaging plane required for LA volume quantification was not available. Nevertheless, Kulka et al. demonstrated that single-plane LA area is reproducible and associated with established cardiovascular risk factors, particularly hypertension and age (19). Similarly, the left atrioventricular coupling index, a promising cardiac MR-derived marker of diastolic dysfunction (36), could not be assessed because LA volumetric measurements were not available. Future studies should evaluate the relationship between the left atrioventricular coupling index and LV filling rates using dedicated cardiac MRI protocols. Traditionally, the assessment of LVDD and HFpEF has heavily relied on invasive catheter-based analysis and operator-dependent echocardiography. Our findings suggest that a non-dedicated whole-body MRI protocol can detect subtle, subclinical alterations in the diastolic axis, potentially identifying subtle cardiopulmonary alterations before overt disease manifestation using a non-invasive, operator-independent imaging modality that is free from ionizing radiation. Whole-body MRI allows simultaneous assessment of cardiac and pulmonary alterations within a single examination, potentially facilitating the detection of early, subclinical disease manifestations, which may facilitate closer monitoring and targeted preventive strategies before overt disease develops.

A limitation of our study is that the development of symptomatic HFpEF in the participants could not be determined due to the study's cross-sectional analysis design. Hence, longitudinal follow-up studies are needed to address this knowledge gap. Due to the study design, invasive measurements and echocardiography were not performed; therefore, only surrogate parameters were available for analysis. LA volume could not be analyzed due to the study protocol. Pulmonary vascular remodeling was assessed using MPA diameter rather than direct hemodynamic measurements. Cardiac biomarkers were not available. The study population was rather small, so even though our study could provide valuable insights into expanding this knowledge, additional research using larger cohorts would be necessary for more robust analyses.

## 5 Conclusion

Population-based whole-body MRI in individuals free from overt cardiovascular disease demonstrated that higher LV filling rates are associated with larger LA and pulmonary vasculature size. Non-invasive, operator-independent whole-body MRI may provide valuable insights into subclinical disease and its progression. Further longitudinal studies to determine the prognostic significance of these findings are necessary.

## Data Availability

Restrictions apply to the availability of the data generated or analyzed during this study to preserve patient confidentiality or because they were used under license. The corresponding author will, on request, detail the restrictions and any conditions under which access to some data may be provided
