# Supplementary material for: Quantitative MRI assessment of left ventricular filling: associations with left atrial and pulmonary artery dimensions in a population-based study
Source: Front Cardiovasc Med. 2026 Aug 5;13:1902569. doi: 10.3389/fcvm.2026.1902569 (PMC13485745; doi:10.3389/fcvm.2026.1902569)
Supplement: Supplementary file 1 [file Datasheet1.docx]

**Supplementary material:**

**Supplementary Table S1- Association between LV early and late diastolic filling rates and the main pulmonary artery-to-ascending aorta ratio in both males and females.**

| Parameter per SD | Model 1 | P value | Model 2 | P value | Model 3 | P value | Model 4 | P value | Model 5 | P value |
| --- | --- | --- | --- | --- | --- | --- | --- | --- | --- | --- |
| **Both genders** | | | | | | | | | | |
| Main pulmonary artery/ascending aorta | | | | | | | | | | |
| LV early diastolic filling rate (ml/s) | 0.02 (0; 0.03) | **0.014** | 0.02 (0; 0.03) | **0.014** | 0.02 (0; 0.04) | **0.018** | 0.02 (0; 0.03) | **0.038** | 0.02 (0; 0.03) | **0.038** |
| LV late diastolic filling rate (ml/s) | 0.01 (0; 0.03) | 0.172 | 0.01 (0; 0.03) | 0.173 | 0.01 (-0.01; 0.02) | 0.207 | 0.01 (-0.01; 0.02) | 0.338 | 0.01 (-0.01; 0.02) | 0.333 |
| MPA/Aa>1 | | | | | | | | | | |
| LV early diastolic filling rate (ml/s) | 1.34 (1.02; 1.77) | **0.036** | 1.34 (1.02; 1.77) | **0.037** | 1.27 (0.95; 1.69) | 0.113 | 1.24 (0.92; 1.66) | 0.159 | 1.24 (0.92; 1.67) | 0.156 |
| LV late diastolic filling rate (ml/s) | 1.05 (0.8; 1.37) | 0.725 | 1.04 (0.8; 1.37) | 0.756 | 1.01 (0.76; 1.34) | 0.958 | 0.97 (0.73; 1.3) | 0.85 | 0.97 (0.72; 1.3) | 0.832 |
| **Females** | | | | | | | | | | |
| Main pulmonary artery/ascending aorta | | | | | | | | | | |
| LV early diastolic filling rate (ml/s) | 0.02 (0; 0.03) | **0.014** | 0.02 (0; 0.03) | **0.014** | 0.02 (0; 0.04) | **0.018** | 0.02 (0; 0.03) | **0.038** | 0.02 (0; 0.03) | **0.038** |
| LV late diastolic filling rate (ml/s) | 0.01 (0; 0.03) | 0.172 | 0.01 (0; 0.03) | 0.173 | 0.01 (-0.01; 0.02) | 0.207 | 0.01 (-0.01; 0.02) | 0.338 | 0.01 (-0.01; 0.02) | 0.333 |
| MPA/Aa>1 | | | | | | | | | | |
| LV early diastolic filling rate (ml/s) | 1.89 (1.23; 2.89) | **0.004** | 1.86 (1.21; 2.86) | **0.005** | 1.63 (1.05; 2.55) | **0.031** | 1.46 (0.92; 2.34) | **0.111** | 1.46 (0.91; 2.34) | 0.113 |
| LV late diastolic filling rate (ml/s) | 1.17 (0.82; 1.67) | 0.388 | 1.15 (0.8; 1.64) | 0.45 | 1.16 (0.79; 1.73) | 0.449 | 1.14 (0.76; 1.7) | 0.524 | 1.16 (0.77; 1.75) | 0.467 |
| **Males** | | | | | | | | | | |
| Main pulmonary artery/ascending aorta | | | | | | | | | | |
| LV early diastolic filling rate (ml/s) | 0.01 (-0.01; 0.02) | 0.447 | 0.01 (-0.01; 0.02) | 0.466 | 0.01 (-0.01; 0.03) | 0.291 | 0.01 (-0.01; 0.03) | 0.364 | 0.01 (-0.01; 0.03) | 0.365 |
| LV late diastolic filling rate (ml/s) | 0.01 (-0.01; 0.03) | 0.345 | 0.01 (-0.01; 0.03) | 0.285 | 0.01 (-0.01; 0.03) | 0.228 | 0.01 (-0.01; 0.02) | 0.462 | 0.01 (-0.01; 0.02) | 0.466 |
| MPA/Aa>1 | | | | | | | | | | |
| LV early diastolic filling rate (ml/s) | 0.99 (0.66; 1.49) | 0.978 | 0.99 (0.66; 1.49) | 0.965 | 1.04 (0.67; 1.63) | 0.862 | 1.04 (0.66; 1.64) | 0.854 | 1.05 (0.66; 1.66) | 0.847 |
| LV late diastolic filling rate (ml/s) | 0.88 (0.54; 1.43) | 0.61 | 0.9 (0.55; 1.45) | 0.657 | 0.92 (0.56; 1.5) | 0.733 | 0.83 (0.51; 1.37) | 0.47 | 0.82 (0.49; 1.36) | 0.434 |

Data are β-coefficients with 95% confidence interval from linear regression models or odds ratios (OR) with 95% confidence interval from logistic regression models

Model 1: Adjusted for age

Model 2: Model 1 + BSA

Model 3: Model 2 + smoking status, hypertension status, diabetes status

Model 4: Model 3 + total cholesterol, triglycerides, hypertension medication, lipid-lowering medication

Model 5: Model 4 + LVEF

Aa – ascending aorta, BSA – body surface area, LA - Left atrium, LAaf - Left atrium area fraction, LAmax - Maximum left atrium area, LAmin - Minimum left atrium area, LA non-gated - Left atrium area derived from axial, non-gated sequences, LDL - Low-density lipoprotein, LV - Left ventricle, LVEF - left ventricular ejection fraction, MPA – Main pulmonary artery

**Table S2- Association between LV early and late diastolic filling rate, LA parameters (a) and pulmonary artery dimensions (b) in females.**

**a.)**

| Parameter per SD | Model 1 | P value | Model 2 | P value | Model 3 | P value | Model 4 | P value | Model 5 | P value |
| --- | --- | --- | --- | --- | --- | --- | --- | --- | --- | --- |
| LAmax (cm^2^) | | | | | | | | | | |
| LV early diastolic filling rate (ml/s) | 1.81 (1.08; 2.54) | **<0.001** | 1.75 (1.03; 2.46) | **<0.001** | 1.75 (1.01; 2.5) | **<0.001** | 1.55 (0.76; 2.34) | **<0.001** | 1.55 (0.76; 2.34) | **<0.001** |
| LV late diastolic filling rate (ml/s) | 0.87 (0.18; 1.56) | **0.014** | 0.79 (0.11; 1.48) | **0.023** | 0.68 (-0.02; 1.39) | 0.057 | 0.56 (-0.13; 1.25) | 0.11 | 0.57 (-0.13; 1.27) | 0.107 |
| LAmin (cm^2^) | | | | | | | | | | |
| LV early diastolic filling rate (ml/s) | 1.17 (0.59; 1.74) | **<0.001** | 1.11 (0.54; 1.67) | **<0.001** | 1.18 (0.6; 1.77) | **<0.001** | 1.06 (0.44; 1.69) | **0.001** | 1.06 (0.43; 1.69) | **0.001** |
| LV late diastolic filling rate (ml/s) | 0.49 (-0.05; 1.03) | 0.073 | 0.43 (-0.11; 0.96) | 0.116 | 0.32 (-0.22; 0.87) | 0.245 | 0.24 (-0.31; 0.78) | 0.393 | 0.24 (-0.31; 0.79) | 0.388 |
| LA area fraction (%) | | | | | | | | | | |
| LV early diastolic filling rate (ml/s) | -0.43 (-2.01; 1.15) | 0.592 | -0.33 (-1.91; 1.24) | 0.678 | -0.75 (-2.36; 0.87) | 0.361 | -0.7 (-2.43; 1.04) | 0.429 | -0.7 (-2.45; 1.04) | 0.426 |
| LV late diastolic filling rate (ml/s) | 0.18 (-1.24; 1.6) | 0.8 | 0.3 (-1.12; 1.71) | 0.679 | 0.5 (-0.93; 1.94) | 0.488 | 0.58 (-0.88; 2.04) | 0.433 | 0.6 (-0.87; 2.07) | 0.422 |
| LA non-gated (cm^2^) | | | | | | | | | | |
| LV early diastolic filling rate (ml/s) | 1.37 (0.74; 2) | **<0.001** | 1.25 (0.67; 1.83) | **<0.001** | 1.32 (0.72; 1.92) | **<0.001** | 1.38 (0.74; 2.01) | **<0.001** | 1.37 (0.73; 2) | **<0.001** |
| LV late diastolic filling rate (ml/s) | 0.76 (0.17; 1.35) | **0.012** | 0.62 (0.08; 1.16) | **0.026** | 0.52 (-0.04; 1.08) | 0.069 | 0.46 (-0.1; 1.03) | 0.107 | 0.48 (-0.08; 1.05) | 0.092 |
| **b.)** | | | | | | | | | | |
| Parameter per SD | Model 1 | P value | Model 2 | P value | Model 3 | P value | Model 4 | P value | Model 5 | P value |
| Main pulmonary artery (cm) | | | | | | | | | | |
| LV early diastolic filling rate (ml/s) | 0.12 (0.05; 0.18) | **<0.001** | 0.11 (0.05; 0.17) | **0.001** | 0.11 (0.05; 0.17) | **<0.001** | 0.11 (0.05; 0.18) | **0.001** | 0.11 (0.05; 0.18) | **0.001** |
| LV late diastolic filling rate (ml/s) | 0.01 (-0.05; 0.07) | 0.744 | 0 (-0.06; 0.05) | 0.911 | 0 (-0.06; 0.05) | 0.905 | -0.01 (-0.07; 0.05) | 0.774 | -0.01 (-0.06; 0.05) | 0.822 |
| Right pulmonary artery (cm) | | | | | | | | | | |
| LV early diastolic filling rate (ml/s) | 0.04 (-0.01; 0.09) | 0.095 | 0.04 (-0.01; 0.08) | 0.142 | 0.03 (-0.02; 0.08) | 0.206 | 0.03 (-0.03; 0.08) | 0.31 | 0.03 (-0.03; 0.08) | 0.298 |
| LV late diastolic filling rate (ml/s) | 0.01 (-0.03; 0.06) | 0.608 | 0 (-0.04; 0.05) | 0.846 | 0.01 (-0.04; 0.05) | 0.787 | 0 (-0.04; 0.05) | 0.883 | 0 (-0.04; 0.05) | 0.937 |
| Left pulmonary artery (cm) | | | | | | | | | | |
| LV early diastolic filling rate (ml/s) | 0.07 (0.03; 0.12) | **0.002** | 0.06 (0.02; 0.1) | **0.002** | 0.07 (0.03; 0.11) | **0.002** | 0.06 (0.01; 0.11) | **0.011** | 0.06 (0.01; 0.11) | **0.011** |
| LV late diastolic filling rate (ml/s) | 0.03 (-0.01; 0.07) | 0.089 | 0.03 (-0.01; 0.06) | 0.17 | 0.03 (-0.01; 0.07) | 0.114 | 0.03 (-0.01; 0.07) | 0.109 | 0.03 (-0.01; 0.07) | 0.103 |

Data are β-coefficients with 95% confidence interval from linear regression models or odds ratios (OR) with 95% confidence interval from logistic regression models

Model 1: Adjusted for age

Model 2: Model 1 + BSA

Model 3: Model 2 + smoking status, hypertension status, diabetes status

Model 4: Model 3 + total cholesterol, triglycerides, hypertension medication, lipid-lowering medication

Model 5: Model 4 + LVEF

BSA – body surface area, LA - Left atrium, LAaf - Left atrium area fraction, LAmax - Maximum left atrium area, LAmin - Minimum left atrium area, LA non-gated - Left atrium area derived from axial, non-gated sequences, LDL - Low-density lipoprotein, LV - Left ventricle, LVEF - left ventricular ejection fraction

**Table S3- Association between LV early and late diastolic filling rate, LA parameters (a) and pulmonary artery dimensions (b) in males.**

**a.)**

| Parameter per SD | Model 1 | P value | Model 2 | P value | Model 3 | P value | Model 4 | P value | Model 5 | P value |
| --- | --- | --- | --- | --- | --- | --- | --- | --- | --- | --- |
| LAmax (cm^2^) | | | | | | | | | | |
| LV early diastolic filling rate (ml/s) | 2.34 (1.74; 2.95) | **<0.001** | 2.36 (1.77; 2.95) | **<0.001** | 2.61 (2; 3.22) | **<0.001** | 2.59 (1.96; 3.21) | **<0.001** | 2.59 (1.96; 3.21) | **<0.001** |
| LV late diastolic filling rate (ml/s) | 1.3 (0.63; 1.96) | **<0.001** | 1.24 (0.59; 1.9) | **<0.001** | 1.22 (0.54; 1.89) | **<0.001** | 1.24 (0.55; 1.93) | **<0.001** | 1.24 (0.55; 1.93) | **0.001** |
| LAmin (cm^2^) | | | | | | | | | | |
| LV early diastolic filling rate (ml/s) | 1.33 (0.86; 1.81) | **<0.001** | 1.35 (0.89; 1.81) | **<0.001** | 1.54 (1.06; 2.02) | **<0.001** | 1.52 (1.03; 2.01) | **<0.001** | 1.52 (1.03; 2.01) | **<0.001** |
| LV late diastolic filling rate (ml/s) | 1.10 (0.61; 1.59) | **<0.001** | 1.05 (0.57; 1.52) | **<0.001** | 1.06 (0.57; 1.55) | **<0.001** | 1.08 (0.58; 1.58) | **<0.001** | 1.1 (0.59; 1.6) | **<0.001** |
| LA area fraction (%) | | | | | | | | | | |
| LV early diastolic filling rate (ml/s) | 0.96 (-0.32; 2.23) | 0.14 | 0.92 (-0.33; 2.18) | 0.148 | 0.8 (-0.53; 2.14) | 0.236 | 0.85 (-0.52; 2.21) | 0.222 | 0.86 (-0.49; 2.21) | 0.211 |
| LV late diastolic filling rate (ml/s) | -0.86 (-2.14; 0.41) | 0.184 | -0.76 (-2.02; 0.5) | 0.237 | -0.94 (-2.23; 0.35) | 0.153 | -0.98 (-2.31; 0.34) | 0.145 | -1.06 (-2.37; 0.26) | 0.114 |
| LA non-gated (cm^2^) | | | | | | | | | | |
| LV early diastolic filling rate (ml/s) | 1.98 (1.49; 2.46) | **<0.001** | 2 (1.54; 2.46) | **<0.001** | 2.15 (1.67; 2.62) | **<0.001** | 2.12 (1.63; 2.61) | **<0.001** | 2.12 (1.63; 2.6) | **<0.001** |
| LV late diastolic filling rate (ml/s) | 1.25 (0.72; 1.79) | **<0.001** | 1.18 (0.66; 1.7) | **<0.001** | 1.17 (0.64; 1.7) | **<0.001** | 1.13 (0.59; 1.68) | **<0.001** | 1.16 (0.62; 1.7) | **<0.001** |
| **b.)** | | | | | | | | | | |
| Parameter per SD | Model 1 | P value | Model 2 | P value | Model 3 | P value | Model 4 | P value | Model 5 | P value |
| Main pulmonary artery (cm) | | | | | | | | | | |
| LV early diastolic filling rate (ml/s) | 0.04 (-0.01; 0.09) | 0.085 | 0.04 (0; 0.09) | 0.078 | 0.04 (-0.01; 0.09) | 0.087 | 0.04 (-0.01; 0.08) | 0.132 | 0.04 (-0.01; 0.08) | 0.134 |
| LV late diastolic filling rate (ml/s) | 0.07 (0.03; 0.12) | **0.002** | 0.07 (0.02; 0.11) | **0.003** | 0.07 (0.02; 0.11) | **0.004** | 0.06 (0.01; 0.1) | **0.013** | 0.06 (0.01; 0.1) | **0.012** |
| Right pulmonary artery (cm) | | | | | | | | | | |
| LV early diastolic filling rate (ml/s) | 0.02 (-0.02; 0.05) | 0.302 | 0.02 (-0.02; 0.05) | 0.277 | 0.03 (-0.01; 0.07) | 0.116 | 0.02 (-0.01; 0.06) | 0.19 | 0.02 (-0.01; 0.06) | 0.193 |
| LV late diastolic filling rate (ml/s) | 0.04 (0; 0.07) | **0.027** | 0.04 (0; 0.07) | **0.037** | 0.04 (0.01; 0.08) | **0.022** | 0.03 (0; 0.07) | 0.076 | 0.03 (0; 0.07) | 0.069 |
| Left pulmonary artery (cm) | | | | | | | | | | |
| LV early diastolic filling rate (ml/s) | 0.07 (0.04; 0.1) | **<0.001** | 0.07 (0.04; 0.1) | **<0.001** | 0.06 (0.03; 0.1) | **<0.001** | 0.06 (0.03; 0.09) | **<0.001** | 0.06 (0.03; 0.09) | **<0.001** |
| LV late diastolic filling rate (ml/s) | 0.07 (0.04; 0.1) | **<0.001** | 0.07 (0.03; 0.1) | **<0.001** | 0.06 (0.03; 0.09) | **<0.001** | 0.05 (0.02; 0.09) | **0.001** | 0.05 (0.02; 0.09) | **0.001** |

Data are β-coefficients with 95% confidence interval from linear regression models or odds ratios (OR) with 95% confidence interval from logistic regression models

Model 1: Adjusted for age

Model 2: Model 1 + BSA

Model 3: Model 2 + smoking status, hypertension status, diabetes status

Model 4: Model 3 + total cholesterol, triglycerides, hypertension medication, lipid-lowering medication

Model 5: Model 4 + LVEF

BSA – body surface area, LA - Left atrium, LAaf - Left atrium area fraction, LAmax - Maximum left atrium area, LAmin - Minimum left atrium area, LA non-gated - Left atrium area derived from axial, non-gated sequences, LDL - Low-density lipoprotein, LV - Left ventricle, LVEF - left ventricular ejection fraction
